# Tracking the ultrafast motion of an antiferromagnetic order parameter

**DOI:** 10.1038/s41467-019-11961-9

**Published:** 2019-09-05

**Authors:** Christian Tzschaschel, Takuya Satoh, Manfred Fiebig

**Affiliations:** 1 Department of Materials, ETH Zurich, 8093 Zurich, Switzerland; 2 Department of Physics, Tokyo Institute of Technology, Tokyo, 152-8551 Japan; 3 Department of Physics, Kyushu University, Fukuoka, 819-0395 Japan

**Keywords:** Spintronics, Nonlinear optics, Magneto-optics

## Abstract

The unique functionalities of antiferromagnets offer promising routes to advance information technology. Their compensated magnetic order leads to spin resonances in the THz-regime, which suggest the possibility to coherently control antiferromagnetic (AFM) devices orders of magnitude faster than traditional electronics. However, the required time resolution, complex sublattice interactions and the relative inaccessibility of the AFM order parameter pose serious challenges to studying AFM spin dynamics. Here, we reveal the temporal evolution of an AFM order parameter directly in the time domain. We modulate the AFM order in hexagonal YMnO_3_ by coherent magnon excitation and track the ensuing motion of the AFM order parameter using time-resolved optical second-harmonic generation. The dynamic symmetry reduction by the moving order parameter allows us to separate electron dynamics from spin dynamics. As transient symmetry reductions are common to coherent excitations, we have a general tool for tracking the ultrafast motion of an AFM order parameter.

## Abstract (web summary)

Understanding antiferromagnetic dynamics enables future information technologies, but the detection remains challenging. Here, the authors show the capability of tracking the three dimensional spin motions in YMnO_3_ by combining time resolved measurements of Faraday rotation and magneto-optical second harmonic generation.

## Introduction

Recently, the appealing properties of antiferromagnets culminated in the demonstration of both optically^1^ and electrically^2^ induced switching of the AFM order. This illustrates the general usefulness of antiferromagnets for future applications^3,4^, yet the dynamics occurring during the switching process remain uncertain as time-resolved (TR) access to the AFM order parameter is notoriously difficult. Consequently, detecting its motion in the time domain often relies on measuring one spin component, such as a transiently occuring uncompensated magnetic moment, and extrapolating the full order-parameter dynamics from models^1^. A detailed study of all spin components during a ferromagnetic spin precession, however, revealed unexpected insights^5,6^. Thus, a dependable analysis of the non-trivial spin dynamics during switching events in antiferromagnets necessitates direct and TR experimental access to all the order-parameter components.

While the uncompensated component is optically accessible by the Faraday effect, detection of the compensated part of the order parameter usually depends on more indirect processes, like magnetic linear birefringence, which is quadratic in the sublattice magnetisation. This leads to a loss of directionality and, thereby, of the ability to distinguish between spin-reversed domain states^2,7–9^. Here, optical second-harmonic generation (SHG), i.e., frequency doubling of a light wave in a material, is a powerful, symmetry-sensitive technique that can couple linearly to the AFM order parameter, thus maintaining its directional information^10,11^. The leading SHG contribution is given by $P_i(2\omega ) = \epsilon _0{\cal{X}}_{ijk}E_j(\omega )E_k(\omega )$, where $\epsilon _0$ is the vacuum permittivity, *E* is the electric field of the incoming light wave, ${\cal{X}}$ is the second-order susceptibility tensor and *P* is the induced polarisation oscillating at twice the frequency *ω* of the incident light^12^. Owing to the direct coupling of ${\cal{X}}$ to the AFM order parameter, SHG is ideal for the static characterisation of antiferromagnets^11,13,14^. Despite this and despite the instantaneous nature of the SHG process, which allows probing ultrafast processes, TR SHG studies of optically induced dynamics are scarce. The methodical difficulty to discriminate between genuine magnetic-order-parameter dynamics, affecting ${\cal{X}}_{ijk}$, and electron dynamics, affecting the linear optical properties at *ω* and 2*ω*, hampers the interpretation of TR SHG measurements^15^. In fact, distinguishing between order-parameter and electron dynamics is commonly believed to be a “practical impossibility”^16^. This is particularly true for thermal excitations, where redistribution of resonantly excited charge carriers leads to substantial changes in the linear optical properties^17–20^.

An optical excitation, however, can also act as a non-thermal stimulus to the magnetic order. A well-known example is the inverse Faraday effect (IFE)^16^, where a circularly polarised laser pulse generates a longitudinal effective magnetic field *H*_IFE_. This field exists for the laser pulse duration only. The impulsive, non-resonant excitation results in a well-defined initial phase of the spin precession. This process is also effective in fully compensated antiferromagnets^8,21–26^. Such a simple fundamental excitation is an ideal test case for us. Its well-understood dynamics allow us to demonstrate how to separate thermal electron dynamics from non-thermal spin dynamics and how to obtain the full order-parameter trajectory.

Here, we use a combination of Faraday rotation and SHG to track the order-parameter motion during a coherent spin precession in antiferromagnetic YMnO_3_. We find that the coherent spin precession transiently reduces the point-group symmetry, which leads to the emergence of new SHG tensor components. We conclude this from a periodic modulation of the SHG anisotropy. In contrast to the Faraday rotation, which is sensitive to the out-of-plane spin component, we show that the SHG modulation reflects the in-plane spin component. Moreover, we quantify both the in-plane and out-of-plane spin-canting angles, where we find a pronounced ellipticity of the spin precession. With this proof-of-concept demonstration, we show that TR SHG is a valuable tool for the study of AFM spin dynamics providing complementary information that is inaccessible by other techniques.

## Results

### Experimental details

We choose hexagonal YMnO_3_ as a model system. The material is ferroelectric below *T*_C_ ≈ 1250 K^27^. The three Mn^3+^ sublattices order antiferromagnetically below the Néel temperature *T*_N_ ≈ 70 K and form a quasi-two dimensional triangular lattice with a fully compensated spin structure. The spins in the sublattices point along the local *x* axes^11^. The magnetic space group of the ground state is $P6_3^\prime cm\prime$, which gives rise to a SHG contribution coupling bilinearly to the ferroelectric and the AFM order parameter^10^ (Supplementary Fig. 1). The magnetic structure yields three orthogonal magnon modes, named *X*-, *Y-* and *Z*-mode, which differ in the direction of the transiently appearing oscillating uncompensated magnetisation component. The individual modes can be excited optically and probed selectively depending on the setting of pump and probe-light polarisations^23^. Here, we exemplarily focus on the *Z*-mode, which has *A*_2_ symmetry^28^.

The optical properties of YMnO_3_ are well-studied^29^. Owing to the hexagonal crystal structure, the material is optically uniaxial, which enables birefringence-free measurements along the z axis. The dielectric spectrum is characterised by a charge-transfer transition around 1.6 eV^30^ and low-optical absorption in the near-infrared regime^31^. In order to minimise parasitic, thermally induced, incoherent dynamics^24^, we optically pump the material at 0.97 eV, where the residual absorption is <10% for our 20 μm thin sample and non-thermal excitation via the IFE dominates. We probe the optically induced dynamics at *ħω* = 1.22 eV, where on the one hand, the high transmittivity enables measurements of the Faraday rotation and on the other hand, efficient SHG is ensured by resonant coupling to a *d–d* transition of the Mn^3+^ ions at 2*ħω*^32^.

**Fig. 1:**
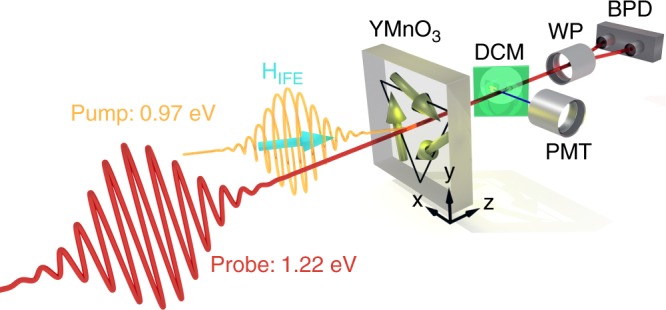
Illustration of the two-colour pump–probe setup. A circularly polarised 130 fs laser pulse at 0.97 eV induces a spin canting in YMnO_3_ via the IFE. The subsequent spin precession is probed with a linearly polarised 1.22 eV laser pulse via a simultaneous measurement of the Faraday rotation (using a Wollaston prism (WP) and a balanced photodiode (BPD)) and the SHG signal (using a Glan–Taylor prism and a photomultiplier tube (PMT), to which the frequency doubled light was branched off by a dichroic mirror (DCM))

Our setup is sketched in Fig. 1. Via the IFE, a circularly polarised pump pulse cants the spins in the *xy* plane by an angle *γμ*_0_*H*_IFE_*τ*, where *γ* is the gyromagnetic ratio, *μ*_0_ is the vacuum permeability and *τ* is the duration of the effective magnetic field pulse^33^. The transmitted linearly polarised probe pulse is split behind the sample by a dichroic mirror for separate, yet simultaneous, TR detection of the Faraday rotation and of the relative change of the SHG intensity (*η* = Δ*I*_SHG_/*I*_SHG_).

### Z-mode observation by Faraday rotation and SHG

**Fig. 2:**
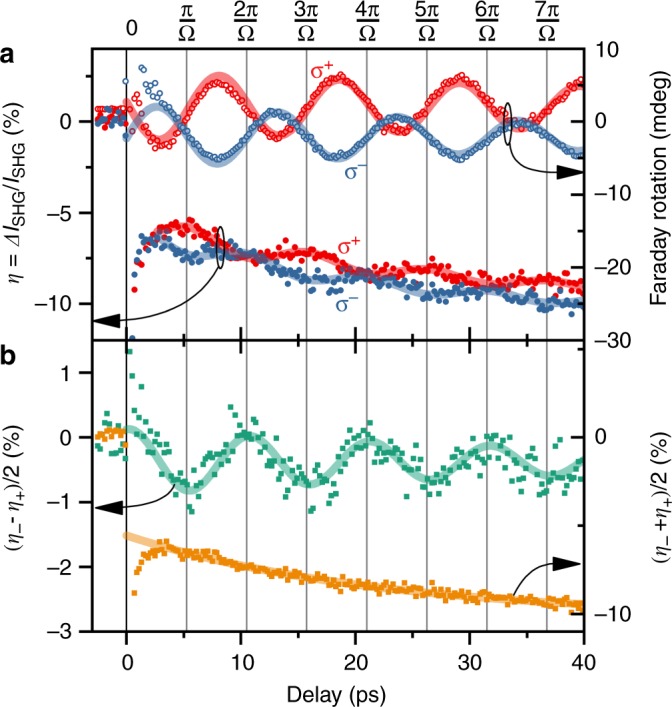
Experimental observation of coherent spin dynamics by Faraday rotation and SHG. Typical measurement of optically induced spin dynamics by *σ*^+^-polarised (red) and *σ*^−^-polarised (blue) pump pulses. **a** Open circles represent Faraday rotation. Closed circles show the relative change of SHG intensity. Solid lines are damped sine (Faraday) and cosine (SHG) fits. **b** Difference (green) and average (orange) of TR SHG changes corroborating helicity-dependent and helicity-independent contributions, respectively. Solid lines represent fits according to a damped cosine (green) and a single exponential decay (orange)

A typical measurement at 10 K is shown in Fig. 2. The Faraday rotation in Fig. 2a exhibits sine-like behaviour with a frequency of Ω/2*π* = 95.2 ± 0.3 GHz. The initial phase changes by *π* upon changing the pump helicity, as is expected for a *Z*-mode excitation via the IFE^23^. In parallel to the Faraday rotation, we measured the SHG response *η* for a probe polarisation enclosing an angle of *ϕ* ≈15° with respect to a crystallographic x axis. We find a coherent modulation of *η* on an incoherent background. By taking (*η*_−_ − *η*_+_)/2 and (*η*_−_ + *η*_+_)/2, with the sign indicating the pump–pulse helicity, we can separate helicity-dependent and helicity-independent contributions, respectively (Fig. 2b). We find that the oscillatory component of *η* is fully contained in the helicity-dependent contribution, while the helicity-independent contribution follows a single exponential decay with a time-constant that is typical for oxide systems^17^. We thus attribute the helicity-independent dynamics to thermalisation processes, in particular also because the quasi-instantaneous effect of possible non-thermal, helicity-independent excitations^34^ cannot explain the exponentially progressing, delayed reduction of SHG intensity in Fig. 2b.

The helicity-dependent SHG difference in Fig. 2b reveals a modulation at the same frequency Ω as the Faraday rotation. We can, therefore, safely conclude that, just like the Faraday rotation, the SHG modulation is based on the optically excited spin precession. Note, however, that the Faraday rotation, which is proportional to the net magnetisation along the z axis, and the SHG modulation are out of phase by *π*/2. Hence, the SHG modulation cannot be based on the out-of-plane spin component. Since the linear transmittance of the sample both at *ω* and 2*ω* does not show any periodic changes either (see Supplementary Fig. 2), we are left with the only option that the SHG modulation reflects the dynamics of the compensated in-plane spin component. We conclude that we observe a modulation of ${\cal{X}}_{ijk}$ induced by dynamic changes of the AFM order parameter $\ell$. There are two fundamentally different mechanisms that can affect ${\cal{X}}_{ijk}$ through $\ell$: (i) symmetry-conserving dynamics involving amplitude modulations of the existing components ${\cal{X}}_{ijk}$. Examples are longitudinal coherent dynamics^35,36^, changes of the exchange interaction^37,38^ as well as thermally induced quenching dynamics^17,20^. Or (ii) symmetry-changing dynamics leading to the appearance of new components ${\cal{X}}_{ijk}$. Besides, e.g., spin-reorientation^39^ and AFM switching processes^1^, this also includes transversal spin dynamics, where precessional spin motion causes a redistribution among the ${\cal{X}}$ components with periodically arising and vanishing contributions.

### TR modulation of the SHG anisotropy

**Fig. 3:**
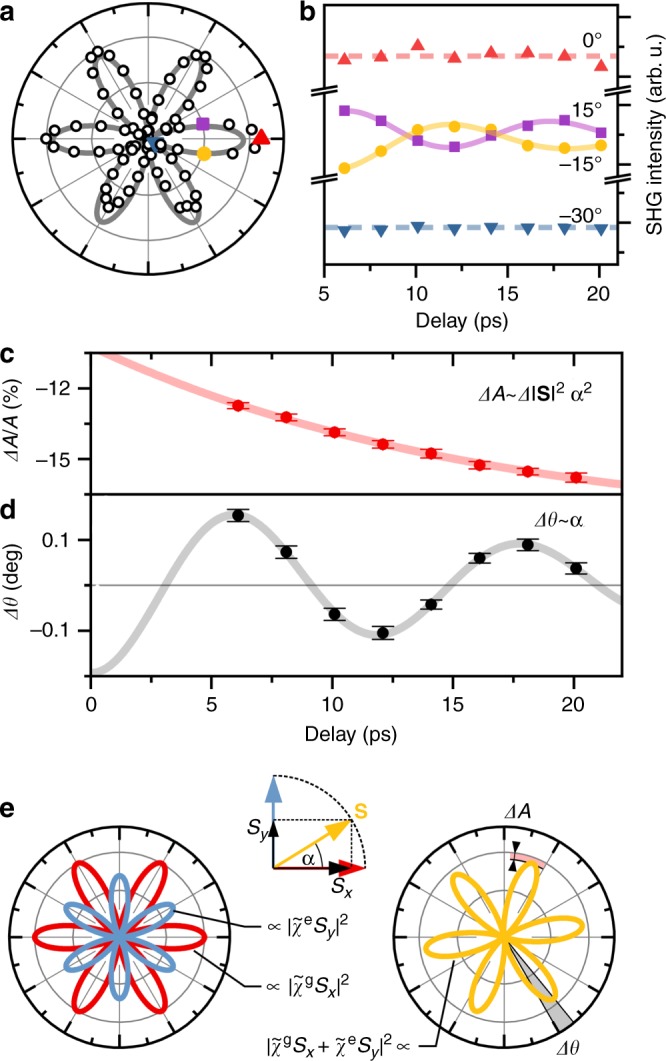
TR modulation of the SHG anisotropy. **a** Measured ground state SHG anisotropy. **b** Time-dependent normalised correlated SHG intensity at different points of the anisotropy (see “Methods”). **c** Time dependence of the relative amplitude change. **d** Time dependent rotation angle of the SHG anisotropy after excitation. Solid lines are fits. The error bars denote standard errors of the means determined from 16 consecutive measurements. **e** Red and blue curves are calculated SHG anisotropies according to $P6_3^\prime cm\prime$ (${\mathbf{S}}\parallel \hat x$) and $P6_3^\prime c\prime m$ (${\mathbf{S}}\parallel \hat y$) symmetry, respectively. A spin canted by an angle *α* leads to a superposition of *S*_*x*_- and *S*_*y*_-related SHG contributions. Their interference causes a rotation and amplitude change of the SHG anisotropy (yellow curve)

We distinguish the two cases by measuring the angle-dependent anisotropy of the SHG signal before and after the excitation. In contrast to Fig. 2, which shows the dynamics of one arbitrary angular component of the SHG anisotropy, measuring the full angle-dependent SHG anisotropy allows us to determine any symmetry changes that might occur. Figure 3a shows the SHG anisotropy prior to excitation. It is in agreement with the $P6_3^\prime cm\prime$ symmetry of the magnetic ground state with the Mn^3+^ spins oriented along equivalent crystallographic x axis. The anisotropy exhibits six lobes of amplitude *A* spaced 60° each. We define their orientation as *θ* = 0°.

The time-dependent SHG intensities at four representative angular components are visualised in Fig. 3b (see “Methods” for details). We find cosine-like modulations with opposite phases at the two sides of a lobe (violet and yellow), but no modulation of the SHG intensity at the minima (blue) or maxima (red). This behaviour cannot be explained by a symmetry-conserving breathing of the SHG anisotropy. Such breathing would manifest itself as an in-phase modulation at the two sides of a lobe and at the maximum. Instead, the observed dynamics are in accordance with a coherent, periodic, small-angle rotation of the SHG anisotropy. By fitting the full SHG anisotropy, we can extract the pump induced changes of the amplitude *A* and the orientation *θ*. The results are shown in Fig. 3c, d, respectively.

The amplitude *A* exhibits an exponential decrease of the order of 10%. The change of *A* cannot be caused by changes of the optical properties as the slight changes of the transmittivity at *ω* and 2*ω* (see Supplementary Fig. 2) are not sufficient to explain the 10% decrease of *A*. Instead, the isotropic, i.e., polarisation-independent, behaviour is indicative of an incoherent relaxation. We, therefore, attribute this decay to longitudinal order-parameter dynamics, i.e. to a reduction of the total sublattice magnetisation gμ_B_|**S**| related to the relaxation of photoexcited charge carriers.

As already indicated by Fig. 3b, d reveals a striking periodic rotation of the SHG anisotropy. The modulation frequency of Δ*θ* matches that of the Faraday rotation, which confirms the magnetic origin. As illustrated in Fig. 3e, a rotated anisotropy can be decomposed into a linear superposition of two sixfold anisotropies at *θ* = 0° (spins along x axis, space group $P6_3^\prime cm\prime$, red curve) and *θ* = 90° (spins along y axis, space group $P6_3^\prime c\prime m$, blue curve). The superposition corresponds to a coherent deviation of the spins from the x axis towards the y axis and, therefore, to a symmetry reduction to $P6_3^\prime$ or lower (see Supplementary Fig. 3). With tan *α* = *S*_*y*_/*S*_*x*_, we can write the dynamic SHG tensor as ${\cal{X}}(t) = \cos \alpha (t)\widetilde {\cal{X}}^g + \sin \alpha (t)\widetilde {\cal{X}}^e$ with time-independent tensors $\widetilde {\cal{X}}^g$ and $\widetilde {\cal{X}}^e$. Thus, complementary to the Faraday rotation, which is sensitive to the uncompensated *z* component of the spin precession, the rotation of the SHG anisotropy reflects the transversal dynamics of the compensated spin component in the *xy* plane. Furthermore, according to the Curie principle^40^, the isotropic effect of a thermal excitation cannot reduce the symmetry of ${\cal{X}}$. Thus, the SHG anisotropy rotation is a pristine measure of the non-thermal spin dynamics. Separating thermal and non-thermal dynamics is therefore possible.

## Discussion

We can use the SHG measurements to quantify the spin deflection angle *α* according to 3Δ*θ* ≈ *ρα* (see Supplementary Note 1), where $\rho = |\widetilde {\cal{X}}^e|/|\widetilde {\cal{X}}^g| = 0.6$ is the amplitude ratio of the real-valued susceptibilities $\widetilde {\cal{X}}_{ijk}$^13,41,42^. Note that *ρ* changes by ±0.3 within the probe laser linewidth, which introduces a systematic error of that order. From Fig. 3d, we extrapolate Δ*θ*(0) = 0.198° ± 0.007° and, hence, *α*(0) = 1.0° with a statistical and systematic uncertainty of 0.035° and 0.5°, respectively. This, in turn, allows us to quantify the effective magnetic field of the IFE as *α*(0) = *γμ*_0_*H*_IFE_*τ*, where 1° corresponds to *μ*_0_*H*_IFE_ ≈760 mT for *τ* = 130 fs (assuming the free electron value *γ* = 176  s^-1^T^-1^). Note that the optically induced spin canting by non-resonant excitation via the IFE is comparable to resonant excitation via the magnetic field component of a strong THz pulse^43^.

In addition to the quantification of the basal-plane spin-canting angle, we can use the TR Faraday rotation to estimate the maximum out-of-plane spin-canting angle as 2.4 mdeg (see Supplementary Note 2). Such a highly anisotropic spin precession is a general phenomenon in antiferromagnets and highlights the dominance of the exchange interaction over the weak magnetic in-plane anisotropy^8,28^.

**Fig. 4:**
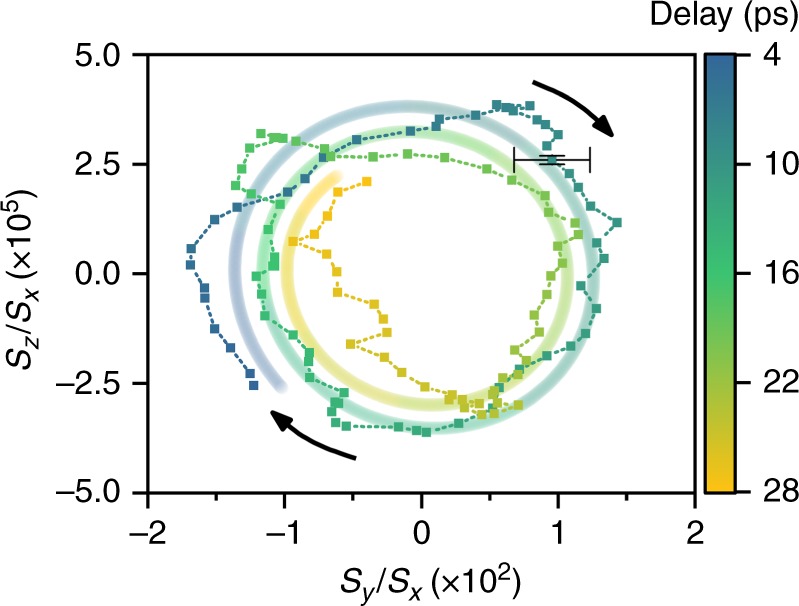
Magnonic *Z*-mode precession. Time dependent amplitude of the spin motion in the *yz* plane obtained by combining floating time averages of SHG and Faraday-rotation measurements. The solid line is a fit of a damped elliptical spin precession. For clarity, only two precession periods are shown

After relating the basal-plane spin-canting angle *α* and the total sublattice magnetisation gμ_B_|**S**| to the SHG anisotropy orientation and amplitude, respectively, we understand the measurement shown in Fig. 2 as follows: The observed TR SHG response is a superposition of longitudinal and transversal spin dynamics and therefore a combination of scenarios (i) and (ii). The time dependence of *α,* and hence *S*_*y*_, is reflected by the periodic SHG modulation. We combine this with the *S*_*z*_-sensitive Faraday rotation to obtain the full order-parameter motion. By plotting the transversal spin components in Fig. 4, one can clearly recognise the magnonic damped elliptical motion (solid line).

We thus present a proof-of-concept experiment, where we track the full three-dimensional motion of an AFM order parameter. In our model compound, YMnO_3_, SHG and Faraday rotation are combined to obtain the trajectory of an optically induced coherent spin precession. While the out-of-plane spin canting induces an uncompensated magnetic moment that causes a Faraday rotation, the basal-plane canting and the total sublattice magnetisation are reflected in the SHG measurements. We quantify the optically induced canting as approximately 1°, corresponding to an effective magnetic field of the IFE of 760 mT. Key to this analysis is not the symmetry of the magnetic ground state, but the transient symmetry reduction during the coherent excitation of an *A*_2_ magnon mode. This symmetry reduction directly affects the highly symmetry-sensitive SHG anisotropy and allows us to separate non-thermal spin dynamics from thermal electron dynamics. We emphasise though that our approach is not at all limited to harmonic small-amplitude dynamics. Instead, we exploit a fundamental excitation to showcase the insights provided by a TR symmetry analysis, while at the same time establishing a general approach for accessing order-parameter dynamics at sub-picosecond timescales. Tracking the full order-parameter motion instead of just one component is indispensable for understanding the highly complex dynamics occurring during ultrafast switching, spin-reorientation and other non-equilibrium phenomena.

## Methods

### Sample

Our YMnO_3_ sample is a single crystal grown by the floating-zone method. We oriented, cut and polished the sample to obtain a 20 μm thin slab with (0001)-oriented surfaces for experiments in transmission geometry. The sample was mounted in a cryostat (Janis SVT-400).

### Pump–probe experimental setup

The fundamental light source of our setup is a regeneratively amplified Ti:Sapphire laser (Coherent, Legend, 2.5 W) producing 130 fs laser pulses at 1 kHz with a central photon energy of 1.55 eV. For our measurements, we used the output of two optical parametric amplifiers providing pump and probe pulses, respectively. To ensure excitation by the IFE, the circularly polarised pump pulses were tuned to 0.97 eV, where the absorption of YMnO_3_ is low. A residual absorption coefficient of ≈30 cm^−1^ at the pump photon energy and ≈40 cm^−1^ at the probe photon energy^31^ results in an absorption of ≈6% and ≈8%, respectively. The pump fluence was approximately 60 mJ cm^−2^. Linearly polarised probe pulses at 1.22 eV allow for the detection of Faraday rotation and SHG. The pump and probe spots exhibit Gaussian profiles with diameters of approximately 180 and 150 μm, respectively. In order to observe the SHG modulation in Fig. 2, the linear polarisation was set to 15° relative to the crystallographic y axis, i.e., to an inclination point of the sixfold antiferromagnetic SHG anisotropy. The transmitted probe pulse passed a dichroic mirror that spatially separated the linear and non-linear optical response. The Faraday rotation was detected using a balanced photodiode, while simultaneously a photomultiplier tube measured the SHG yield in the pumped and unpumped state.

### SHG anisotropy measurements

All measurements were performed in near-normal incidence. The polarisation dependence of the SHG anisotropy was obtained by keeping the sample fixed and rotating the light polarisation of the incoming probe laser pulse with a half-wave plate. The transmission direction of a Glan-Laser prism inserted between the dichroic mirror and the photomultiplier tube (not shown in Fig. 1) was always set perpendicular to the polarisation of the incoming probe light. Each SHG anisotropy consists of 73 individual data points spaced by 5°. The changes of amplitude Δ*A*/*A* and orientation Δ*θ* were extracted from a fit according to

1 $$I(\phi ) = C + A\cos ^23\left( {\phi + \theta } \right){,}$$

The uncertainties shown in Fig. 3c, d denote standard errors of the amplitudes and orientations.

Figure 3b is obtained by normalising the SHG anisotropy for each delay time to its maximum, averaging each data point with its symmetry equivalent points, i.e.,

2 $$I_{{\mathrm{ave}}}(\phi ) = \frac{1}{6}\mathop {\sum}\limits_{i = 0}^5 I (\phi + i \ast 60^ \circ ){,}$$

and plotting *I*_ave_(0°), *I*_ave_(±15°) and *I*_ave_(−30°).

## Supplementary information

Supplementary Information

Peer Review File

## Data Availability

The data that support the findings of this study are available from the corresponding author upon reasonable request.
