# Supplementary material for: Tracking the ultrafast motion of an antiferromagnetic order parameter
Source: Nat Commun. 2019 Sep 5;10:3995. doi: 10.1038/s41467-019-11961-9 (PMC6728322; doi:10.1038/s41467-019-11961-9)
Supplement: Supplementary file 1 — Supplementary Information [file 41467_2019_11961_MOESM1_ESM.pdf]

Supplementary Information

Tracking the ultrafast motion of an antiferromagnetic order parameter

Ch. Tzschaschel *et al.*

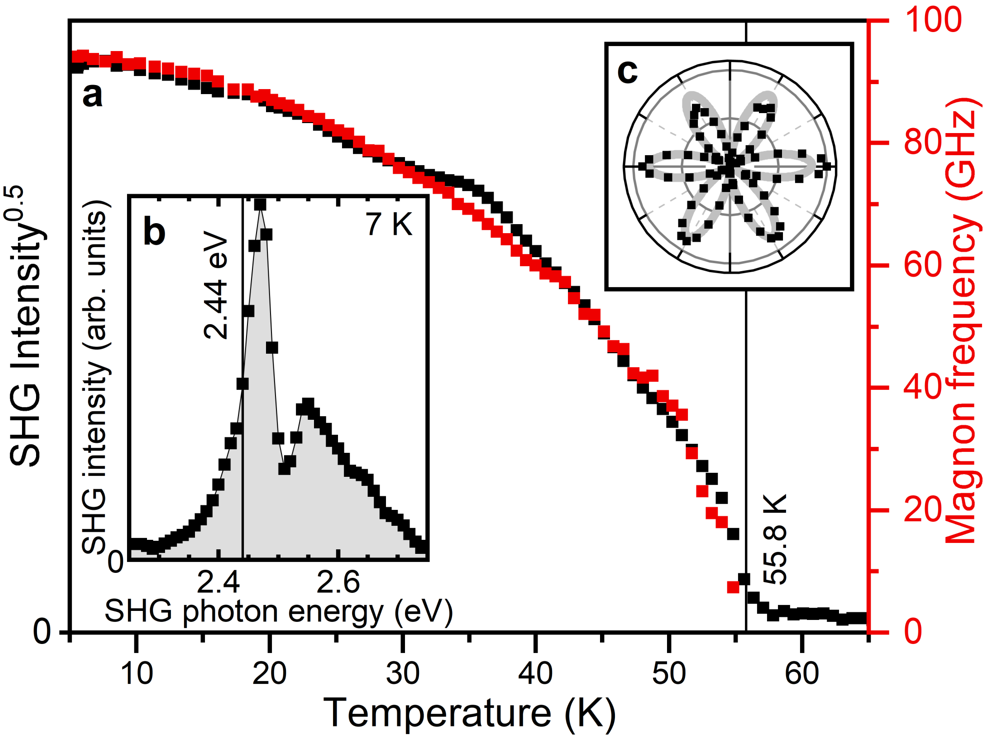

Supplementary Fig. 1: Coupling of $\mathcal{X}_{ijk}$ to AFM order parameter a Temperature-dependence of the magnon frequency and the total SHG intensity. Black squares correspond to the total SHG yield; red squares to the extracted magnon frequency. Both measures show a critical behaviour around 56 K, which we identify as the Néel-temperature. The discrepancy between the measured transition temperature and literature values is presumably based on laser induced heating and sample variations. **b** Measured SHG spectrum at 7 K. It agrees nicely with literature [1]. An electronic transition from the $^5\Gamma_1$ ground state to a $^5\Gamma_6$ state at 2ω causes a resonant enhancement of the SHG intensity. The high transparency at the fundamental probe photon energy allows to perform all measurements in transmission geometry [2, 3]. The probe photon energy was set to 2.44 eV(SHG) for all measurements. **c** Sixfold SHG anisotropy of the magnetic ground state reflecting the $P6_3'cm'$ symmetry.

The magnon frequency is proportional to the sublattice magnetisation $\omega \propto |\mathbf{S}|$ [4, 5], which is proportional to the antiferromagnetic order parameter ℓ. In addition, previous SHG measurements were explained assuming a bilinear coupling of $\mathcal{X}_{ijk}$ to $\mathbf{P} \cdot \ell$ ($YMnO_3$ is ferroelectric below 1270 K with polarisation $\mathbf{P} \parallel \hat{z}$.) [6]. The identical temperature dependence of the SHG intensity ($\propto \mathcal{X}_{ijk}^2$) and the magnon frequency is proof that changes of $\mathbf{P}$ are negligibly small in the considered temperature range. Hence, the SHG effectively probes exclusively the antiferromagnetic order parameter ℓ and $\mathcal{X}_{ijk} \propto \ell$. Therefore, our approach for tracking the antiferromagnetic order parameter is also applicable to non-polar antiferromagnets.

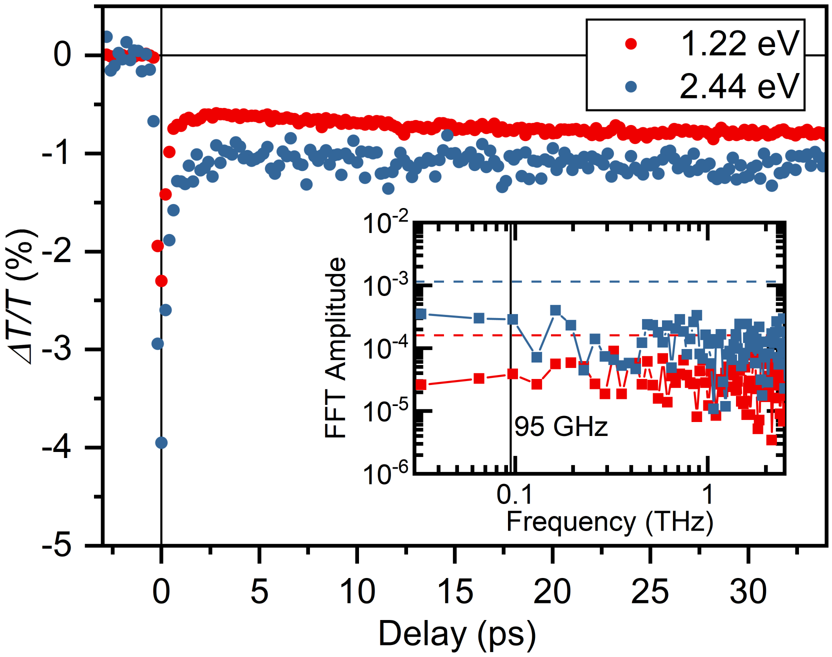

Supplementary Fig. 2: Optically induced transmission changes Optically induced transmission changes at the fundamental (red) and second-harmonic (blue) frequency. Inset: FFT for delays $\geq$2.8 ps after substracting an exponentially decaying offset. Dashed lines indicate the respective noise level estimated from the standard deviation of the data for delays ≤ -0.6 ps.

We are not able to detect any periodic modulation of the transmission at neither the fundamental nor second-harmonic frequency. As the amplitude of the observed modulation (Fig. 2 in the main text) exceeds the noise level by at least one order of magnitude, we can confidently attribute it to periodic changes of $\mathcal{X}_{ijk}$.

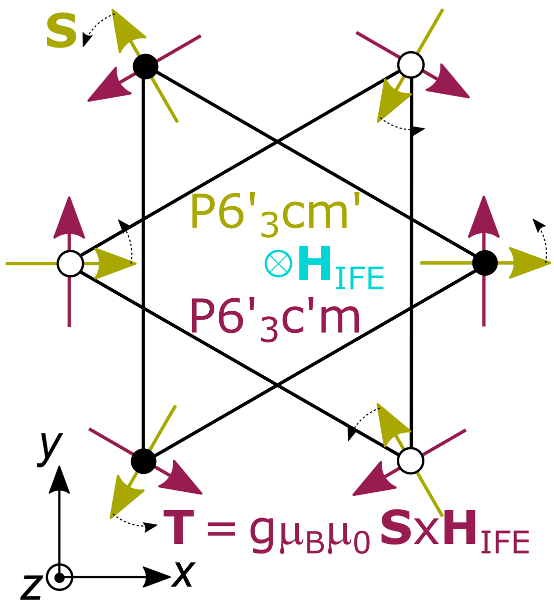

Supplementary Fig. 3: Derivation of excited state symmetry in h-YMnO$_3$ The ground state spin arrangement is illustrated by yellow arrows in Fig. 3. The magnetic space group is $P6_3'cm'$. The IFE acts as an effective magnetic field pulse $\mathbf{H}_{\mathrm{IFE}}$ along the z axis, which results in an in-plane torque $\mathbf{T} = g\mu_B\mu_0\mathbf{S}\times\mathbf{H}_{\mathrm{IFE}}$ (indicated by red arrows). The resulting torque field has the symmetry $P6_3'c'm$ and leads to a spin canting along the black arrows. The magnetic space group immediately after the optical excitation is the intersection of the ground state symmetry and the torque field symmetry, i.e. $P6_3'$. After excitation, the effective magnetic field points along the spins' equilibrium positions, which leads to a spin precession around the equilibrium state. The corresponding torque field has the symmetry $P3c'1$ reducing the symmetry to $P3$, which is in general the magnetic space group during the Z mode precession.
Consequently, the linear optical properties of the sample for light propagating along the z-axis are isotropic, even during the spin precession [7]. Therefore, linear optical techniques that relay on a magnetically induced optical anisotropy to provide information about the in-plane spin dynamics, such as magnetic linear dichroism [8], magnetic linear birefringence [9] or longitudinal MOKE [10] are not effective.

Supplementary Note 1. Influence of the relative phase between $\tilde{\mathcal{X}}^0$ and $\tilde{\mathcal{X}}^e$

We will show in this section, how a relative phase factor between the ground state SHG contributions $\tilde{\mathcal{X}}^0$ and the excited state contributions $\tilde{\mathcal{X}}^e$ will affect the pump induced changes of the SHG anisotropy. We therefore consider a linearly polarised laser pulse propagating along the z axis. The electric field can be written as

$$\mathbf{E} = E_0 \begin{pmatrix} \cos\phi \\ \sin\phi \end{pmatrix}$$

This induces a polarisation

$$P_i(2\omega) = \epsilon_0 \mathcal{X}_{ijk} E_j E_k,$$

which acts as a source for the emitted SHG intensity. The allowed SHG contributions during the Z-mode excitation are $\mathcal{X}^0_{yyy} = -\mathcal{X}^0_{xxy} = -\mathcal{X}^0_{xyx} = -\mathcal{X}^0_{yxx}$ and $\mathcal{X}^e_{xxx} = -\mathcal{X}^e_{yyx} = -\mathcal{X}^e_{yxy} = -\mathcal{X}^e_{xyy}$ [7].

We detect the SHG intensity perpendicular to the incoming laser field, i.e. in the direction

$$\mathbf{A} = \begin{pmatrix} \sin\phi \\ -\cos\phi \end{pmatrix}$$

The detected SHG intensity is then

$$I(\phi) = |\mathbf{P} \cdot \mathbf{A}|^2 \tag{1}$$
$$= |\mathcal{X}^0_{yyy} \cos 3\phi - \mathcal{X}^e_{xxx} \sin 3\phi|^2 I_0^2 \tag{2}$$
$$= \left(|\mathcal{X}^0_{yyy}|^2 \cos^2 3\phi + |\mathcal{X}^e_{xxx}|^2 \sin^2 3\phi - \Re\left\{\mathcal{X}^e_{xxx} \bar{\mathcal{X}}^0_{yyy}\right\} \sin 6\phi\right) I_0^2 \tag{3}$$

As $\mathcal{X}_{yyy}$ and $\mathcal{X}_{xxx}$ couple linearly to the x and y component of the sublattice magnetisation [11], respectively, we can relate the measured anisotropy to the basal plane spin canting angle α by writing

$$\mathcal{X}^0_{yyy} = \tilde{\mathcal{X}}^0_{yyy} \cos\alpha \tag{4}$$

$$\mathcal{X}^e_{xxx} = \tilde{\mathcal{X}}^e_{xxx} \sin\alpha \tag{5}$$

This is justified by the pronounced magnetic anisotropy [4], which leads to a strongly elliptical spin precession. Note that $\tilde{\mathcal{X}}^0_{yyy}$ and $\tilde{\mathcal{X}}^e_{xxx}$ are complex quantities and thus characterized by an amplitude and a phase. Static measurements of hexagonal rare-earth manganites that show a spin reorientation transition indicate an amplitude ratio $\rho = |\tilde{\mathcal{X}}^e|/|\tilde{\mathcal{X}}^0| \lesssim 1$ [12, 13], but give no information on the relative phase ψ. However, the SHG anisotropy has to reflect the threefold symmetry in accordance with the $P3$ symmetry of the excited state and can thus in general be written in the form

$$I(\phi) = C + A\cos^2(3\phi + \delta)$$

with (for $\alpha \ll 1$):

$$C \approx I_0^2 |\tilde{\mathcal{X}}^0_{yyy}|^2 \rho^2 \alpha^2 \sin^2\psi \tag{6}$$

$$A \approx I_0^2 |\tilde{\mathcal{X}}^0_{yyy}|^2 \left(1 - \alpha^2 + \rho^2\alpha^2 \cos 2\psi\right) \tag{7}$$

$$\delta \approx \rho\alpha\cos\psi \tag{8}$$

It is worth noting that only the SHG anisotropy rotation Eq. (8) is linear in α, while Eqs. (6) and (7) are quadratic. Symmetry-based measurements are therefore intrinsically more susceptible to the subtle changes of the spin order as compared to intensity based measurements in any minimum or maximum of the SHG anisotropy. Furthermore, as Eqs. (6) and (7) are even functions of α, positive and negative spin canting angles cannot be distinguished, leading to an SHG modulation at twice the magnon frequency. The extreme cases of $\psi = 0$ and $\psi = \pi/2$ are illustrated by the calculated SHG anisotropies in Fig. 4. For clarity, exaggerated spin canting angles of $\pm 10°$ are shown together with the ground-state orientation of $0°$.

As Eqn. (8) depends on the ratio ρ of the contributing SHG tensor components, it is crucial to avoid cases, where $\rho = 0$. Specific to the hexagonal manganites, the $\mathcal{X}^e_{xxx}$ component of the

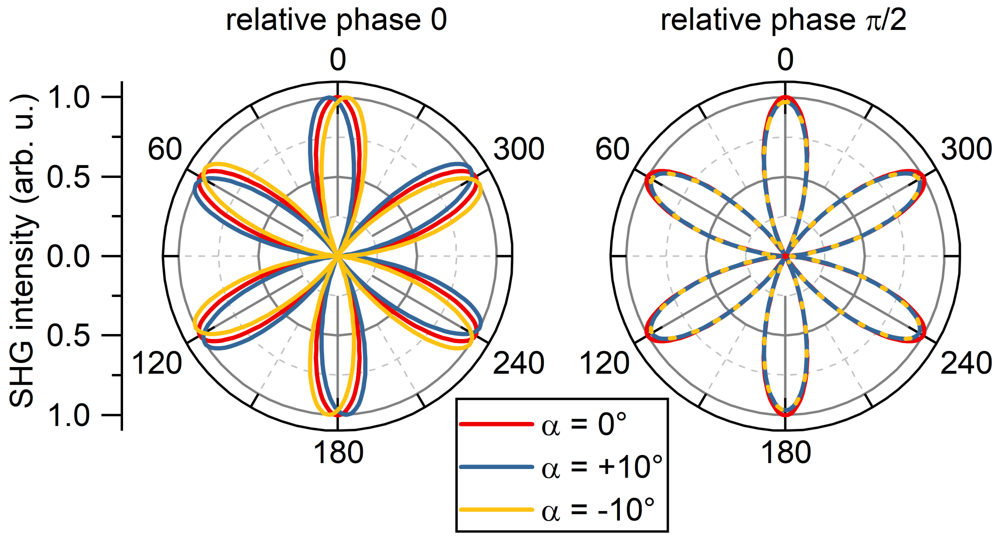

Supplementary Fig. 4: Calculated SHG anisotropies Left: vanishing relative phase ψ between $\tilde{\mathcal{X}}^0_{yyy}$ and $\tilde{\mathcal{X}}^e_{xxx}$. Right: relative phase of $\psi = \pi/2$.

excited state vanishes at the maximum of the ground state contribution $\mathcal{X}^0_{yyy}$, leading to $\rho = 0$ [14, 15]. Therefore, we performed all measurements slightly off resonant at 2.44 eV (SHG) (cf. Supplementary Fig. 1b).

Supplementary Note 2. Determination of out-of-plane spin canting angle ϕ_z

We will show in this section, how we arrive at an out-of-plane spin canting angle ϕ_z of approximately 2.4 mdeg.

An exponentially damped sine fit of the time-resolved Faraday rotation shown in Fig. 2a in the main text reveals an amplitude of ≈ 3.3 mdeg. As $YMnO_3$ and $ErMnO_3$ exhibit very similar Verdet constants [16], we can use the Verdet spectrum of $ErMnO_3$ [1] to relate the measured Faraday rotation to a magnetic field. In particular, we find a Verdet constant of 53.7 deg T^{-1} mm^{-1} at the probe photon energy of 1.22 eV. A Faraday rotation of 3.3 mdeg thus corresponds to a magnetic field of $\mu_0 H_z \approx$ 3.1 mT for a sample thickness of 20 μm.

We use the following Hamiltonian to describe the magnetic system:

$$\mathcal{H} = J \sum_{\langle i,j \rangle} \mathbf{S}_i \cdot \mathbf{S}_j + D_z \sum_i S_{i,z}^2 - g\mu_B \mu_0 H_z \sum_i S_{i,z} \tag{9}$$

with the Landé factor $g \approx 1.9$ [17], $J \approx$ 2.45 meV, $D_z \approx$ 0.48 meV [18] and μ_B the Bohr magneton. The static solution to the Landau-Lifshitz equation then yields:

$$S_z \approx \frac{g\mu_B \mu_0 H_z}{3J + 2D_z} \approx 4.1 \cdot 10^{-5},$$

which corresponds to the above mentioned out-of-plane spin canting of 41 μrad = 2.4 mdeg.

Supplementary references

References

[1] Degenhardt, C., Fiebig, M., Fröhlich, D., Lottermoser, Th. & Pisarev, R. V. Nonlinear optical spectroscopy of electronic transitions in hexagonal manganites. *Appl. Phys. B* **73,** 139-144 (2001).

[2] Babonas, G.-J., Grivel, J.-C., Reza, A. & Girkantaite, R. Ellipsometric studies of $ErMnO_3$ single crystals. *Lith. J. Phys.* **47,** 309–314 (2007).

[3] Wang, Y. T., Luo, C. W. & Kobayashi, T. Understanding multiferroic hexagonal manganites by static and ultrafast optical spectroscopy. *Adv. Condens. Matter Phys.* **2013** 104806 (2013).

[4] Sato, T. J. *et al.* Unconventional spin fluctuations in the hexagonal antiferromagnet $YMnO_3$. *Phys. Rev. B* **68,** 014432 (2003).

[5] Vajk, O. P., Kenzelmann, M., Lynn, J. W., Kim, S. B. & Cheong, S.-W. Magnetic Order and Spin Dynamics in Ferroelectric $HoMnO_3$. *Phys. Rev. Lett.* **94,** 087601 (2005).

[6] Fiebig, M., Lottermoser, Th., Fröhlich, D., Goltsev, A. V. & Pisarev, R. V. Observation of coupled magnetic and electric domains. *Nature* **419,** 818 (2002).

[7] Birss, R. R. *Symmetry and Magnetism* (North Holland Publishing Company, Amsterdam, 1966).

[8] Tesařová, N. *et al.* Direct measurement of the three-dimensional magnetization vector trajectory in GaMnAs by a magneto-optical pump-and-probe method. *Appl. Phys. Lett.* **100,** 102403 (2012).

[9] Saidl, V. *et al.* Optical determination of the Néel vector in a CuMnAs thin-film antiferromagnet. *Nat. Photonics* **11,** 91–96 (2017).

[10] Keatley, P. S. *et al.* Time- and vector-resolved magneto-optical Kerr effect measurements of large angle precessional reorientation in a $2{\times}2\,\mu m^2$ ferromagnet. *J. Appl. Phys.* **105** 07D308 (2009).

[11] Sa, D., Valentí, R. & Gros, C. A generalized Ginzburg-Landau approach to second harmonic generation. *Eur. Phys. J. B* **14,** 301–305 (2000).

[12] Fiebig, M., Fröhlich, D., Lottermoser, Th. & Kohn, K. Spin-angle topography of hexagonal manganizes by magnetic second-harmonic generation. *Appl. Phys. Lett.* **77,** 4401 (2000).

[13] Lottermoser, Th. & Fiebig, M. Magnetoelectric behavior of domain walls in multiferroic $HoMnO_3$. *Phys. Rev. B* **70,** 220407(R) (2004).

[14] Fiebig, M. *et al.* Determination of the Magnetic Symmetry of Hexagonal Manganites by Second Harmonic Generation. *Phys. Rev. Lett.* **84,** 5620–5623 (2000).

[15] Fiebig, M., Pavlov, V. V. & Pisarev, R. V. Second-harmonic generation as a tool for studying electronic and magnetic structures of crystals: review. *J. Opt. Soc. Am. B* **22,** 96–118 (2005).

[16] Fiebig, M., Degenhardt, C. & Pisarev, R. V. Interaction of frustrated magnetic sublattices in $ErMnO_3$. *Phys. Rev. Lett.* **88,** 027203 (2002).

[17] Standard, E. C., Stanislavchuk, T., Sirenko, A. A., Lee, N. & Cheong, S.-W. Magnons and crystal-field transitions in hexagonal RMnO$_3$ (R = Er, Tm, Yb, Lu) single crystals. *Phys. Rev. B* **85,** 144422 (2012).

[18] Toulouse, C. *et al.* Lattice and spin excitations in multiferroic h-$YMnO_3$. *Phys. Rev. B* **89,** 094415 (2014).
