# Supplementary material for: Tracking the ultrafast motion of an antiferromagnetic order parameter
Source: Nat Commun. 2019 Sep 5;10:3995. doi: 10.1038/s41467-019-11961-9 (PMC6728322; doi:10.1038/s41467-019-11961-9)
Supplement: Supplementary file 2 — Peer Review File [file 41467_2019_11961_MOESM2_ESM.pdf]

Reviewers' comments:

Reviewer #1 (Remarks to the Author):

The manuscript entitled “Tracking the ultrafast motion of an antiferromagnetic order parameter” by Christian Tzschaschel et al presents the time resolved spin precession dynamics in a hexagonal antiferromagnet of $YMnO_3$ excited by ultrafast laser pulses. The authors have tried to demonstrate, by combining the techniques of time resolved second harmonic generation (SHG) and magneto-optical Faraday effect, the capability of tracking the three dimensional spin motions in $YMnO_3$ with triangular spin orientations. I agree that the detection of the dynamics of antiferromagetic (AFM) order parameter is a very challenging issue, in particular for using the table top facilities. Though very recently there were some reports on the detection of the dynamics of AFM order parameter, for example in CuMnAs (already cited as a reference) and CoO with collinear spin arrangement, using time resolved magnetic linear dichroism effect with table top ultrafast lasers, how to track the dynamical AFM order parameter in triangular spin lattice and also with three dimensional trajectory is still left as an open question. In this sense, the approach of tracking the AFM order parameter suggested by the authors in this manuscript may greatly advance the field of the AFM spin dynamics. However, there are some major issues the authors need to clarify before I can recommend the publication of this manuscript in Nature Communications.

1) The authors did not present a direct measurement of the time evolution of the anisotropic SHG pattern, which is actually a key evidence to directly demonstrate the AFM order’s periodic oscillation in the x-y plane. The periodic changes of $\Delta\theta$ and SHG amplitude A shown in Fig. 3 seem to be just obtained from the analysis and model calculations based on the measured data of Fig. 2. I suggest that the transient SHG intensities should at least be detected on the two sides of one of the six lobes of the anisotropic SHG pattern to show the opposite phases of their oscillations. This result can give more direct evidence of the in-plane oscillation of the AFM spin direction.
2) The oscillating out-of-plane spin component may also contribute to the transient SHG signals. The authors should clarify its contributions (including the frequency and phase) due to the corresponding change of the symmetry group to distinguish them from the measured SHG signals.

Also, some minor issues:
3) No indication of how to obtain the anisotropic SHG patters was given in the main manuscript. Does it correspond to the rotation of the sample?
4) On page 4, line 82, it was written as “The helicity-dependent difference reveals cosine-like behaviour with a frequency matching that of the Faraday rotation. The initial phase reflects the helicity dependence of the excitation mechanism, albeit with a phase shift of $\pi/2$ with respect to the Faraday response. Therefore, the SHG modulation cannot be related to the transient uncompensated magnetization component.” This is not a straightforward case for the readers who are not familiar with the SHG technique. Better to revise it. This point is actually related to the above point 2.

Reviewer #2 (Remarks to the Author):

This is a very interesting and well-written paper that convincingly demonstrates the utility of using time-resolved SHG anisotropy to map changes in antiferromagnetic order in complex oxide materials. These results will be of significant interest to the broader quantum materials community.
I recommend publication of this paper, but suggest that the following comments be addressed in order to clarify and strengthen the impact.

The paper is lacking details (and basic references) on the optical properties of YMnO_3. While the inset in figure S3 indicates the photon energy dependence of the SHG signal, it would be good to put this in context of the basic optical properties and relevant optical transitions of the material. There is a

rather cryptic comment in the Supplemental Information about avoiding the complications that might occur at resonance. This warrants a bit more discussion. How do the observed effects depend on the probe photon energy?

Similarly, how do the effects depend on the excitation photon energy? At a minimum, one might expect that the ratio between the thermal (incoherent) dynamics and the non-thermal spin dynamics would depend critically on how close the excitation photon energy is to the absorption resonance (apparently at ~1.5 eV in YMnO_3). An additional dependence might be expected for excitation photon energies that are less than half the absorption resonance (where two-photon absorption effects might be suppressed).
What is the optical density (optical thickenss) of the sample at the pump and probe wavelengths?

It seems that complementary information could be obtained via polar/vector time-resolved MOKE methods. A brief mention (and reference) would help put the present work in context.

Two additional minor points:

A reference to the Curie principle would be useful to add.

Line 106: Total sublattice magnetization |S| should be defined here

Reviewer #3 (Remarks to the Author):

In this manuscript, the authors introduce a method to track the temporal dynamics of spins in antiferromagnetic materials. Finding efficient ways to trace the ultrafast dynamics of order parameters in antiferromagnetic materials is an important topic in rapidly growing field of antiferromagnetic spintronics. The present paper proposes a novel method for temporal tracking of 3D dynamics of order parameter in antiferromagnets and thus is interesting for researchers in the community. I think the manuscript satisfies the criteria for publication in Nature Communications.
In the following, I have listed a few comments and questions which I believe the authors should address them before publishing the manuscript:
1) I am wondering if this method can also be applied for collinear antiferromagnets. What about non-ferroelectric antiferromagnetic materials?
2) It is not clear to me if helicity independent contribution is necessarily related to thermal process. In a few recent experimental works like, Nat. Commun. 6, 8190 (2015), and theoretical works, arXiv:1904.01270 and Nat. Commun. 6, 6708 (2015) possibility of atheraml and helicity independent spin excitation have been discussed.
3) In the Supplementary Information, it has been written that “it is well known that the magnon frequency couples linearly to the sublattice magnetization”. I do not understand this sentence. What does mean the linear coupling of magnon frequency and sublattice magnetization? Can authors explain that or at least write its formula?

Responses to reviewers' comments

Reviewer #1 (1)

The manuscript entitled "Tracking the ultrafast motion of an antiferromagnetic order parameter" by Christian Tzschaschel et al presents the time resolved spin precession dynamics in a hexagonal antiferromagnet of YMnO3 excited by ultrafast laser pulses. The authors have tried to demonstrate, by combining the techniques of time resolved second harmonic generation (SHG) and magneto-optical Faraday effect, the capability of tracking the three dimensional spin motions in YMnO3 with triangular spin orientations. I agree that the detection of the dynamics of antiferromagetic (AFM) order parameter is a very challenging issue, in particular for using the table top facilities. Though very recently there were some reports on the detection of the dynamics of AFM order parameter, for example in CuMnAs (already cited as a reference) and CoO with collinear spin arrangement, using time resolved magnetic linear dichroism effect with table top ultrafast lasers, how to track the dynamical AFM order parameter in triangular spin lattice and also with three dimensional trajectory is still left as an open question. In this sense, the approach of tracking the AFM order parameter suggested by the authors in this manuscript may greatly advance the field of the AFM spin dynamics.

We thank the reviewer for this precise summary. We are happy that the reviewer shares our view on the importance of tracking the AFM order parameter.

Reviewer #1 (2)

However, there are some major issues the authors need to clarify before I can recommend the publication of this manuscript in Nature Communications.

1) The authors did not present a direct measurement of the time evolution of the anisotropic SHG pattern, which is actually a key evidence to directly demonstrate the AFM order's periodic oscillation in the x-y plane. The periodic changes of Δθ and SHG amplitude A shown in Fig. 3 seem to be just obtained from the analysis and model calculations based on the measured data of Fig. 2. I suggest that the transient SHG intensities should at least be detected on the two sides of one of the six lobes of the anisotropic SHG pattern to show the opposite phases of their oscillations. This result can give more direct evidence of the in-plane oscillation of the AFM spin direction.

We apologise for creating the impression that Fig. 3 was a result obtained from the data shown in Fig. 2. In fact, Fig. 3 is based on a measurement of the full 360° SHG anisotropy, just as the reviewer suggests. As such it already contains part of the results requested by the reviewer. We now provide an additional figure showing the full analysis described by the reviewer (Fig. 3a and b). Indeed, we observe oscillations of opposite phases on the two sides of a lobe.

We discuss that figure in lines 121-129 in the main text and clarify the independence of the two data sets in Figs. 2 and 3 in lines 115-117 in the main text.

Reviewer #1 (3)

2) The oscillating out-of-plane spin component may also contribute to the transient SHG signals. The authors should clarify its contributions (including the frequency and phase) due to the corresponding change of the symmetry group to distinguish them from the measured SHG signals.

We agree with the reviewer. An out-of-plane spin component S_z reduces the symmetry of the magnetic ground state. This may lead to (i) new components of the SHG tensor and (ii) a modification of existing components. With light propagating along the *z* axis, we cannot couple to the newly allowed tensor components of (i). Regarding (ii), the modification would be proportional to the

longitudinal net magnetisation. This has two implications. On the one hand, the expected modulation would be in phase with the Faraday rotation, while we observe the SHG modulation and Faraday rotation to be out of phase. On the other hand, the already existing components are proportional to $|S|$. Thus, the expected modulation depth would be roughly of the order of $S_z/|S| < 10^{-4}$ and therefore too small to explain the observation. This allows us to conclude that we are not probing the out-of-plane spin component with SHG.

We clarified this distinction in lines 98-104.

Reviewer #1 (4)
Also, some minor issues:
3) No indication of how to obtain the anisotropic SHG patters was given in the main manuscript. Does it correspond to the rotation of the sample?

We now explain how the anisotropy data were obtained in the methods section (lines 310-321).

Reviewer #1 (5)
4) On page 4, line 82, it was written as "The helicity-dependent difference reveals cosine-like behaviour with a frequency matching that of the Faraday rotation. The initial phase reflects the helicity dependence of the excitation mechanism, albeit with a phase shift of π/2 with respect to the Faraday response. Therefore, the SHG modulation cannot be related to the transient uncompensated magnetization component." This is not a straightforward case for the readers who are not familiar with the SHG technique. Better to revise it. This point is actually related to the above point 2.

The inverse Faraday effects creates a magnetic-field pulse in the material, whose direction depends on the helicity of the incident light. This magnetic field induces a spin precession that causes an oscillating net magnetisation, and hence a Faraday rotation with a sine-like time-dependence. The helicity dependence of the magnetic field direction is reflected in the initial phase of the spin precession. As the modulation of the SHG is also helicity-dependent and exhibits the same frequency as the modulation of the Faraday rotation, we conclude that the SHG modulation is based on the optically excited spin precession. The SHG modulation and the Faraday rotation are, however, by $\pi/2$ out of phase. Hence, the SHG modulation cannot be based on the out-of-plane spin component. This leaves us with the only option that the SHG modulation reflects the in-plane component of the spin precession.

We included this explanation in lines 88-104.

Reviewer #2 (1)
This is a very interesting and well-written paper that convincingly demonstrates the utility of using time-resolved SHG anisotropy to map changes in antiferromagnetic order in complex oxide materials. These results will be of significant interest to the broader quantum materials community.
I recommend publication of this paper, but suggest that the following comments be addressed in order to clarify and strengthen the impact.

We thank the reviewer for working on our manuscript and this appreciative comment.

Reviewer #2 (2)
The paper is lacking details (and basic references) on the optical properties of YMnO_3. While the inset in figure S3 indicates the photon energy dependence of the SHG signal, it would be good to put this in context of the basic optical properties and relevant optical transitions of the material. There is a rather cryptic comment in the Supplemental Information about avoiding the complications that might occur at resonance. This warrants a bit more discussion. How do the observed effects depend on the probe photon energy?

We added the following references regarding the optical properties of hexagonal manganites to the main text (cited in lines 69-77) and to the Supplementary Information.

- For a general review of the optical properties: Wang et al. (2013, now Ref. 28)
- For absorption data: Kalashnikova et al. (2003, now Ref. 29)
- For a detailed near-IR absorption spectrum: Babonas et al. (2007, now Ref. 30)
- For an electronic level diagram: Degenhardt et al. (2001, now Ref. 31)

Regarding the complications on resonance: This is not a general problem, but specific to the hexagonal manganites. The modulation of the SHG anisotropy is based on interference between the SHG components corresponding to $\boldsymbol{S} \parallel \boldsymbol{x}$ and $\boldsymbol{S} \parallel \boldsymbol{y}$. For the hexagonal manganites, the spectral component of the ($\boldsymbol{S} \parallel \boldsymbol{y}$)-SHG vanishes at the resonance of the ($\boldsymbol{S} \parallel \boldsymbol{x}$)-SHG (as shown in Refs. [S10] and [S11]) making it impossible to observe the required interference. We thus measured slightly off resonant.

We extended the discussion of the resonance effects in the Supplementary Information, Section 3, to clarify this.

Reviewer #2 (3)
Similarly, how do the effects depend on the excitation photon energy? At a minimum, one might expect that the ratio between the thermal (incoherent) dynamics and the non-thermal spin dynamics would depend critically on how close the excitation photon energy is to the absorption resonance (apparently at ~1.5 eV in YMnO_3). An additional dependence might be expected for excitation photon energies that are less than half the absorption resonance (where two-photon absorption effects might be suppressed).

Indeed, in earlier measurements, we changed the pump photon energy from 0.97 eV to 1.37 eV, i.e. closer to the absorption resonance at 1.6 eV. We found a significant increase in the incoherent background as the residual absorption in the tail of the resonance gets more pronounced. It is therefore crucial to stay as far below 1.6 eV as possible. By shifting below 0.97 eV, in particular below 0.8 eV, however, the output power of the OPA deteriorates. 0.97 eV therefore constitutes the optimum for us.

With reference to a detailed study by Bossini et al. (Ref. 23), we now mention in line 72-74 that we excited the spin dynamics at 0.97 eV to minimise the thermally induced, incoherent background signal.

Reviewer #2 (4)
What is the optical density (optical thickenss) of the sample at the pump and probe wavelengths?

The optical absorption spectrum is dominated by a charge-transfer transition at around 1.6 eV. Thus, the sample is transparent at both the pump photon energy of 0.97 eV and the probe photon energy of 1.22 eV. As reported by Babonas et al. (Ref 27 in the main text), typical values for the absorption coefficient are <40 cm^{-1} resulting in an internal transmission of >90% for our sample. As mentioned in the response to Reviewer #2 (3), this is sufficient to ensure the non-thermal spin excitation to be dominant.

We now explicitly mention the absorption at the pump- and probe wavelength in the methods section (lines 300-301).

Reviewer #2 (5)
It seems that complementary information could be obtained via polar/vector time-resolved MOKE methods. A brief mention (and reference) would help put the present work in context.

In contrast to ferromagnets, however, the net-magnetization during the Z-mode precession occurs solely along the Z-axis. Thus, the oscillating spin component would only show up in polar MOKE. Polar MOKE and Faraday rotation measurements provide equivalent information about the out-of-plane spin component. However, for transparent samples Faraday rotation provides larger rotation angles. As $YMnO_3$ is transparent at the probe photon energy, we decided for Faraday rotation measurements.

Exemplarily, for an order parameter tracking in ferromagnets, a reference to Tesarova et al. (2012, now Ref. 6) has been added to the main text (line 29). Furthermore, we now discuss conditions for the applicability of equivalent linear optical techniques in our case in Supplementary Information, section 2.

Reviewer #2 (6)
Two additional minor points:

A reference to the Curie principle would be useful to add.

A reference to the textbook *Crystal Symmetries* by Hargittai and Vainshtein has been added to both the main text (Ref. 39) and the Supplementary Information (Ref. S1).

Reviewer #2 (7)
Line 106: Total sublattice magnetization |S| should be defined here

We followed the reviewer's comment.

Reviewer #3 (1)

In this manuscript, the authors introduce a method to track the temporal dynamics of spins in antiferromagnetic materials. Finding efficient ways to trace the ultrafast dynamics of order parameters in antiferromagnetic materials is an important topic in rapidly growing field of antiferromagnetic spintronics. The present paper proposes a novel method for temporal tracking of 3D dynamics of order parameter in antiferromagnets and thus is interesting for researchers in the community. I think the manuscript satisfies the criteria for publication in Nature Communications. In the following, I have listed a few comments and questions which I believe the authors should address them before publishing the manuscript:

We thank the reviewer for the work invested into reading our manuscript and are glad about its positive reception.

Reviewer #3 (2)

1) I am wondering if this method can also be applied for collinear antiferromagnets. What about non-ferroelectric antiferromagnetic materials?

Yes, this is in general possible. Second-harmonic generation is a symmetry-sensitive technique that is particularly suitable to detect non-centrosymmetry in materials, but it also sensitive to other types of symmetry breaking. Since any type of ferroic order breaks point-group symmetries (see e.g. V.K. Wadhawan, *Introduction to Ferroic Materials*), SHG can, in principle, detect any type of ferroic transition, including the examples mentioned by the Reviewer.

We now state in the Supplementary Information that our method can also be applied to non-polar antiferromagnets.

Reviewer #3 (3)

2) It is not clear to me if helicity independent contribution is necessarily related to thermal process. In a few recent experimental works like, Nat. Commun. 6, 8190 (2015), and theoretical works, arXiv:1904.01270 and Nat. Commun. 6, 6708 (2015) possibility of atheraml and helicity independent spin excitation have been discussed.

Indeed, there are non-thermal excitation mechanisms for coherent spin dynamics that are not sensitive to the pump helicity, such as optically induced modifications of exchange interactions or magnetic anisotropies. These effects are, however, based on the presence of the laser pulse and get weaker with increasing delay time. Contrarily, the helicity-independent reduction of SHG intensity that we observe is most pronounced for the largest delay times. The delayed response together with the exponentially progressing signal is indicative for thermal effects. A thermally induced, partial quenching of the order parameter thus provides a plausible explanation for the exponentially decaying, helicity-independent contribution to our signal.

We adapted the main text in lines 91-96 according to this discussion.

Reviewer #3 (4)

3) In the Supplementary Information, it has been written that “it is well known that the magnon frequency couples linearly to the sublattice magnetization”. I do not understand this sentence. What does mean the linear coupling of magnon frequency and sublattice magnetization? Can authors explain that or at least write its formula?

The magnon frequency is determined by the exchange interaction and the magnetic anisotropy of the lattice (see e.g. Kittel, *Introduction to Solid State Physics*). As for any antiferromagnet, the spin lattice can be separated into several ferromagnetic sublattices. The exchange field and the anisotropy field are proportional to the sublattice magnetisation $|S|$, which is up to a factor equal to the ordered magnetic moment per lattice site. In the specific case of $YMnO_3$, it was shown by e.g. Sato et al. or Vajk et al. (Refs. S12 and S13 in the Supplementary Information) that the specific formula depends on the magnon mode, but the proportionality $\omega{\sim}|S|$ always holds.

We rephrased that sentence and added the general proportionality to the Supplementary Information.

REVIEWERS' COMMENTS:

Reviewer #1 (Remarks to the Author):

The authors have addressed my previous questions and concerns. But for the completeness of the introduction to the time resolved laser spectroscopy on studying the antiferromagnetic (AFM) spin order, I suggest the authors to include in the introduction part one more reference of Phys. Rev. B 98, 134409 (2018), in which the laser induced AFM spin dynamics of CoO films using quadratic magneto-optical spectra was demonstrated (as I mentioned in the last referee report). This work and the paper reporting the AFM spin dynamics in CuMnAs films prove the capability of the time resolved quadratic magneto-optical spectra to study the spin dynamics in various types of compensated AFM films.
I recommend the publication of the manuscript in Nature Communications after the minor revision.

Reviewer #3 (Remarks to the Author):

The authors have answered all questions and comments by details. They have also revised the manuscript based on the reviewers suggestions. I believe the present version of the manuscript satisfies the Nat. Commun. criteria and thus it is ready for publication.

Responses to reviewers‘ comments

Reviewer #1 (6)

The authors have addressed my previous questions and concerns. But for the completeness of the introduction to the time resolved laser spectroscopy on studying the antiferromagnetic (AFM) spin order, I suggest the authors to include in the introduction part one more reference of Phys. Rev. B 98, 134409 (2018), in which the laser induced AFM spin dynamics of CoO films using quadratic magneto-optical spectra was demonstrated (as I mentioned in the last referee report). This work and the paper reporting the AFM spin dynamics in CuMnAs films prove the capability of the time resolved quadratic magneto-optical spectra to study the spin dynamics in various types of compensated AFM films.
I recommend the publication of the manuscript in Nature Communications after the minor revision.

We included the suggested reference and are grateful to the reviewer for recommending our manuscript for publication.

Reviewer #3 (5)

The authors have answered all questions and comments by details. They have also revised the manuscript based on the reviewers suggestions. I believe the present version of the manuscript satisfies the Nat. Commun. criteria and thus it is ready for publication.

We thank the reviewer for the appreciative comment and for recommending our manuscript for publication.
